# Accurate Identification of *ALK* Positive Lung Carcinoma Patients: Novel FDA-Cleared Automated Fluorescence *In Situ* Hybridization Scanning System and Ultrasensitive Immunohistochemistry

**DOI:** 10.1371/journal.pone.0107200

**Published:** 2014-09-23

**Authors:** Esther Conde, Ana Suárez-Gauthier, Amparo Benito, Pilar Garrido, Rosario García-Campelo, Michele Biscuola, Luis Paz-Ares, David Hardisson, Javier de Castro, M. Carmen Camacho, Delvys Rodriguez-Abreu, Ihab Abdulkader, Josep Ramirez, Noemí Reguart, Marta Salido, Lara Pijuán, Edurne Arriola, Julián Sanz, Victoria Folgueras, Noemí Villanueva, Javier Gómez-Román, Manuel Hidalgo, Fernando López-Ríos

**Affiliations:** 1 Laboratorio de Dianas Terapéuticas, Centro Integral Oncológico “Clara Campal”, Hospital Universitario Madrid Sanchinarro, Universidad San Pablo-CEU, Madrid, Spain; 2 Hospital Ramón y Cajal, Madrid, Spain; 3 C.H.U. A Coruña, La Coruña, Spain; 4 Hospital Virgen del Rocío, Sevilla, Spain; 5 IdiPAZ (Hospital La Paz Institute for Health Research), University Hospital La Paz, Faculty of Medicine, Autonomous University of Madrid, Madrid, Spain; 6 Hospital Insular de Gran Canaria, Las Palmas de Gran Canaria, Spain; 7 C.H.U. Santiago de Compostela, Santiago de Compostela, Spain; 8 Hospital Clinic, Barcelona, Spain; 9 Hospital del Mar-Parc de Salut Mar, Barcelona, Spain; 10 Hospital Clínico San Carlos, Madrid, Spain; 11 Hospital Central de Asturias, Oviedo, Spain; 12 Hospital Marqués de Valdecilla, Santander, Spain; 13 Oncology Department, Centro Integral Oncológico “Clara Campal”, Hospital Universitario Madrid Sanchinarro, Universidad San Pablo-CEU, Madrid, Spain; Istituto dei tumori Fondazione Pascale, Italy

## Abstract

**Background:**

Based on the excellent results of the clinical trials with *ALK*-inhibitors, the importance of accurately identifying *ALK* positive lung cancer has never been greater. However, there are increasing number of recent publications addressing discordances between FISH and IHC. The controversy is further fuelled by the different regulatory approvals. This situation prompted us to investigate two ALK IHC antibodies (using a novel ultrasensitive detection-amplification kit) and an automated *ALK* FISH scanning system (FDA-cleared) in a series of non-small cell lung cancer tumor samples.

**Methods:**

Forty-seven *ALK* FISH-positive and 56 *ALK* FISH-negative NSCLC samples were studied. All specimens were screened for ALK expression by two IHC antibodies (clone 5A4 from Novocastra and clone D5F3 from Ventana) and for *ALK* rearrangement by FISH (Vysis ALK FISH break-apart kit), which was automatically captured and scored by using Bioview's automated scanning system.

**Results:**

All positive cases with the IHC antibodies were FISH-positive. There was only one IHC-negative case with both antibodies which showed a FISH-positive result. The overall sensitivity and specificity of the IHC in comparison with FISH were 98% and 100%, respectively.

**Conclusions:**

The specificity of these ultrasensitive IHC assays may obviate the need for FISH confirmation in positive IHC cases. However, the likelihood of false negative IHC results strengthens the case for FISH testing, at least in some situations.

## Introduction

In August 2011, crizotinib, a novel *ALK* tyrosine kinase inhibitor, was approved by the US FDA for the treatment of patients with locally advanced or metastatic non-small-cell lung carcinomas (NSCLCs) that are *ALK*-positive as detected by an FDA-approved test (i.e. Vysis ALK FISH Break-Apart Probe Kit) [1]. Soon afterwards, the drug was approved by the EMA, with the statement that “an accurate and validated *ALK* assay is necessary for the selection of patients” [2]. Based on these excellent results of the crizotinib clinical trials and the development of other *ALK* inhibitors with consistent efficacy results in this patient population, the importance of accurately identifying *ALK* positive lung cancer has never been greater [3].

Few areas in cancer biomarkers have been as contentious as *HER2* testing in breast cancer patients. Since 1998, we have witnessed a huge clinical advance in this field and, however, a great biomarker conundrum over methods, cut-off points, and algorithms [immunohistochemistry (IHC) *versus* fluorescence *in situ* hybridization (FISH) as the primary testing assay] [4], [5]. The outcome is a significant percentage of false negative (12%) or false positive results (14%) [6].

This controversy is also entering the field of NSCLC *ALK* testing [7], with an increasing number of recent publications addressing discordances between *in situ* hybridization and IHC assays [8]–[14], further fuelled by the different regulatory approvals and the arrival of other *ALK* inhibitors [3], [15]. While some groups recommend initial IHC followed by FISH confirmation of some IHC-positive cases [14], [16], others believe the detection of *ALK* rearrangements is improved when using two methodologies [9], [17]. This situation prompted us to investigate two IHC antibodies, using a novel ultrasensitive detection-amplification kit, and an automated FISH scanning system in a series of tumor samples to obtain supporting data for an *ALK* testing algorithm [18]. To our knowledge, there has not been an independent assessment of *ALK* concordance between these three assays using our strategy (i.e., FDA-cleared automated FISH scanning system) in a large series of *ALK* positive tumors.

## Material and Methods

### Tumor samples

Seventy-nine *ALK* FISH-positive samples from patients with advanced NSCLCs procured at 11 hospitals were used for this study. The Institutional Ethics Committee at Grupo Hospital de Madrid reviewed and approved this study and waived the need for consent. Samples were consecutive *ALK* positive cases, initially tested as part of routine clinical care. In addition, 77 consecutive *ALK* FISH-negative samples from advanced NSCLCs diagnosed at the referral institution were included as negative controls. The material available for all tumors had been formalin-fixed and paraffin-embedded (FFPE). The specifics of formalin fixation were unknown. All cases were classified by two pathologists (E.C. and F.L-R.) [19], [20]. All specimens were independently screened for ALK expression by two IHC antibodies, and for *ALK* rearrangement by FISH, which was scored using an automated scanning system (FDA-cleared) [21]. Cases were excluded if we could not score a minimum of 50 nuclei (i.e., gold standard package insert recommendation, see below). The Institutional Ethics Committee at the referral institution reviewed and approved this study.

### FISH for *ALK* rearrangement

FISH was performed on unstained 4 µm-thick FFPE tumor tissue sections using the *ALK* break-apart probe set (Vysis ALK FISH break-apart kit; Abbott Molecular, IL, USA), following the manufacturer's instructions [22], [23]. The *ALK* FISH assay was independently captured and scored with the automated BioView Duet scanning system (BioView, Rehovot, Israel) by two pathologists blinded to the IHC results (E.C. and A.S-G.). The system included a fluorescent microscope (Olympus), a high-resolution progressive-scan charge-coupled device digital camera, and a computer equipped with imaging and analysis software. The procedure consisted of the following steps: (1) proper tumour tissue sections were selected for automated imaging and analysis using a ×10 objective to locate the nuclei; (2) the system automatically captured and analyzed the nuclei found in those regions using a ×60 objective with immersion oil and the single band DAPI/SpectrumGreen/SpectrumOrange filter; and (3) the system recorded and classified each target nuclei utilizing a specific algorithm of positive or negative signal patterns based upon the classifications described in the Vysis *ALK* FISH break-apart kit product insert enumeration instructions (also used in the crizotinib clinical trials). Nuclei that the system could not match to defined signal patterns were placed in the unclassified category.

A minimum of 50 tumor nuclei were counted. *ALK* FISH-positive cases were defined as more than 25 (50%) break-apart (BA) signals or an isolated signal (IRS) in tumor cells. *ALK* FISH-negative samples were defined as less than 5 (10%) BA or IRS cells. *ALK* FISH cases were considered borderline if 5–25 (10–50%) cells were positive. In the case of borderline results, a second reader evaluated the slide, added cell count readings from the already automatically captured images, and a percentage was calculated out of 100 cells. If the positive cells percentage was lower than 15%, the sample was considered negative. If the positive cells percentage was higher or equal to 15%, the sample was considered positive (refer to the package insert for Vysis ALK Break Apart FISH Probe Kit, Cat. No. 06N38-020/30-608495/R2).

### IHC for ALK expression

Automated IHC for ALK expression was performed for all cases in a Benchmark XT staining module (Ventana Medical Systems, Tucson, AZ). FFPE tumor tissues were sectioned at a thickness of 4 µm and stained with two different ALK antibodies: Ventana anti-ALK rabbit monoclonal primary antibody (Clone D5F3, Ventana Medical Systems, Tucson, AZ), and Novocastra mouse monoclonal antibody p80 ALK (Clone 5A4, Novocastra, Newcastle, United Kingdom). Briefly, the Ventana anti-ALK antibody was applied with OptiView DAB IHC Detection Kit and OptiView Amplification Kit, performing one serial tissue section for Ventana anti-ALK (D5F3), and a second serial tissue section for a Rabbit Monoclonal Negative Control Ig antibody, following the manufacturer's instructions. The Novocastra (5A4) antibody was used at 1∶20 dilution, treated, and incubated at 37°C for 2 hours. Detection was performed with the same OptiView detection-amplification kit. FISH-validated *ALK*-positive and *ALK*-negative external controls were included in all the slides.

The slides were reviewed by two pathologists (E.C. and F.L-R.) blinded to FISH results. The results of both ALK IHC assays were evaluated using a modified H-score: strong cytoplasmic staining (3+), clearly visible using a ×2 or ×4 objective; moderate staining (2+), requiring a ×10 or ×20 objective to be clearly seen; and weak staining (1+), cannot be seen until a ×40 objective is used [21]. Both anti-ALK IHC staining results were interpreted using a binary scoring system: positive (3+ or 2+) or negative (1+ or 0), adapting to the manufacturer's instructions [refer to the package insert for Ventana anti-ALK (D5F3) Rabbit Monoclonal Primary Antibody, Cat. No. 790-4794/06679072001] and in agreement with recently released survival data in crizotinib treated patients [24].

### Statistical data analysis

Based on all the valid data obtained, we performed a descriptive analysis of both the independent and dependent variables of interest. This analysis was stratified by specimen type, location and histologic type. The technique used for comparison of frequencies was Pearson's χ2 test (frequency <5, Fisher). The normality of the continuous variables was verified using the Kolmogorov-Smirnov test. As these variables, i.e. number of positive cells and number of negative cells, did not follow a normal distribution, non-parametric tests were used. For comparison of means we used the Kruskal-Wallis test. The sensitivity, specificity, and positive and negative predictive values of the Ventana anti-ALK, Novocastra (5A4), and FISH using an automated scoring system were obtained. Statistical differences were deemed significant at p <0.05. Statistical data analyses were performed using the Statistical Package for Social Sciences (version 19.0; Chicago, IL, USA).

## Results

The results are summarized in Table 1.

**Table 1:** Concordance between ALK IHC and *ALK* FISH.

ALK IHC		*ALK* FISH			Sensitivity (%) (95% CI)	Specificity (%) (95% CI)	PPV (%) (95% CI)	NPV (%) (95% CI)	Accuracy (%) (95% CI)
		FISH+	FISH−	Total (%)					
**IHC Ventana (D5F3)**									
IHC+	3+	46	0	46 (44.7)	98 (95–100)	100 (100–100)	100 (100–100)	98 (96–100)	99 (97–100)
	2+	0	0						
IHC−	1+	0	8	57 (55.3)					
	0	1	48						
Total (%)		47 (45.6)	56 (54.4)	103 (100)					
**IHC Novocastra (5A4)**									
IHC+	3+	41	0	46 (44.7)	98 (95–100)	100 (100–100)	100 (100–100)	98 (96–100)	99 (97–100)
	2+	5	0						
IHC−	1+	0	0	57 (55.3)					
	0	1	56						
Total (%)		47 (45.6)	56 (54.4)	103 (100)					

*IHC, immunohistochemistry; FISH, fluorescence in situ hybridization; CI, confidence interval; PPV, positive predictive value; NPV, negative predictive value.*

### *ALK* rearrangement assessed by FISH

**Figure 1:**
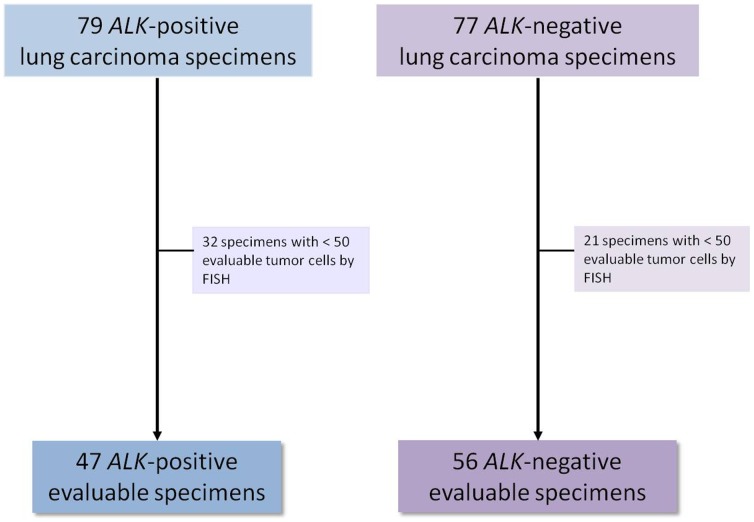
Study design and specimen selection.

Of the 79 *ALK*-positive lung carcinoma specimens, 32 cases were excluded for lack of tumor tissue. Of the 77 *ALK*-negative NSCLCs, 21 specimens were excluded for lack of tumor tissue (Figure 1). Among the 103 available cases analyzed, 47 tumors (45.6%) had an *ALK* rearrangement, showing the two major described patterns [BA pattern in 21.3% of cases (10/47), IRS pattern in 44.7% of tumors (21/47), and both patterns in 34% of tumors (16/47)]. Fifty-six (54.4%) cases were negative, showing two fusion signals or very close green and red signals. The total number of tumor cells analyzed was 50 in 98 cases (95.1%) and 100 in 5 specimens (4.9%) (cases with initial borderline results). In *ALK* FISH-negative cases, the mean percentage of positive cancer cells was 0.7% (median 0%; range 0 to 6%). In *ALK* FISH-positive tumors, the mean percentage of positive cells was 68.2% (median 68%; range 25 to 94%). In three of these *ALK*-rearranged cases, the percentage of positive cells was less than 50% (25%, 36% and 46%, respectively). Among FISH *ALK*-positive cases, we observed 5 tumors (10.6%) with *ALK* amplification, as previously described [25].

### Correlation between ALK IHC and *ALK* FISH data

**Figure 2:**
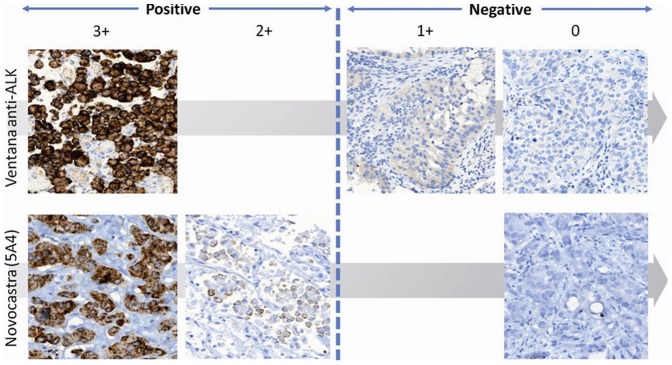
Immunostaining pattern of ALK in NSCLC using Ventana anti-ALK (D5F3) and Novocastra (5A4) antibodies. ALK IHC reveals variable levels of protein expression: from absent (0) to weak/faint cytoplasmic staining (1+) in negative cases and from moderate (2+) to strong (3+) granular cytoplasmic immunstaining in positive tumors. In ALK IHC-negative cases, the immunoreactivity was always 0 by Novocastra (5A4) IHC, whereas ranged from 0 to 1+ by Ventana antibody. However, in ALK IHC-positive cases, protein expression was always 3+ by Ventana antibody, whereas it ranged from 2+ to 3+ by Novocastra (5A4) IHC. Original magnification: 400×.

All cases with IHC scores of 3+ (strong cytoplasmic staining) by Ventana anti-ALK antibody, and all cases with IHC scores of 2+ and 3+ by Novocastra (5A4) antibody (ALK IHC-positive cases) were FISH-positive. All cases but one with IHC scores of 1+ and 0 by Ventana, and with IHC scores of 0 by Novocastra (ALK IHC-negative cases) were FISH-negative (Figure 2). There was only one IHC-negative case with both antibodies which showed a FISH-positive result (IRS-rearranged pattern in an average of 84% of tumor cells). Additional blocks were requested and re-tested with identical results (data not shown). Interestingly, it was a surgically resected (lobectomy), poorly differentiated squamous cell carcinoma (SCC) (i.e., p40 positive by IHC, data not shown). Given the discrepancy, all results were independently reviewed (F.L-R.) and confirmed.

### ALK immunoreactivity by IHC

Following the above criteria, among the 103 available cases analyzed, 46 cases (44.7%) were positive, whereas 57 tumors (55.3%) were negative by both Ventana anti-ALK and Novocastra (5A4) antibodies. Interestingly, in ALK IHC-negative cases, the immunoreactivity was always absent (0) by Novocastra (5A4) IHC, whereas it ranged from absent to weak/faint cytoplasmic staining (1+) by Ventana antibody. However, in ALK IHC-positive cases, protein expression was always strong cytoplasmic staining (3+) by Ventana anti-ALK antibody, whereas it ranged from moderate (2+) (n = 5) to strong staining (3+) (n = 41) by Novocastra (5A4) IHC (Figure 2). In 15 positive cases (32.6%) by Ventana IHC and 16 positive tumors (34.8%) by Novocastra IHC, we noted significant intratumoral heterogeneity, ranging from weak to strong protein expression.

**Figure 3:**
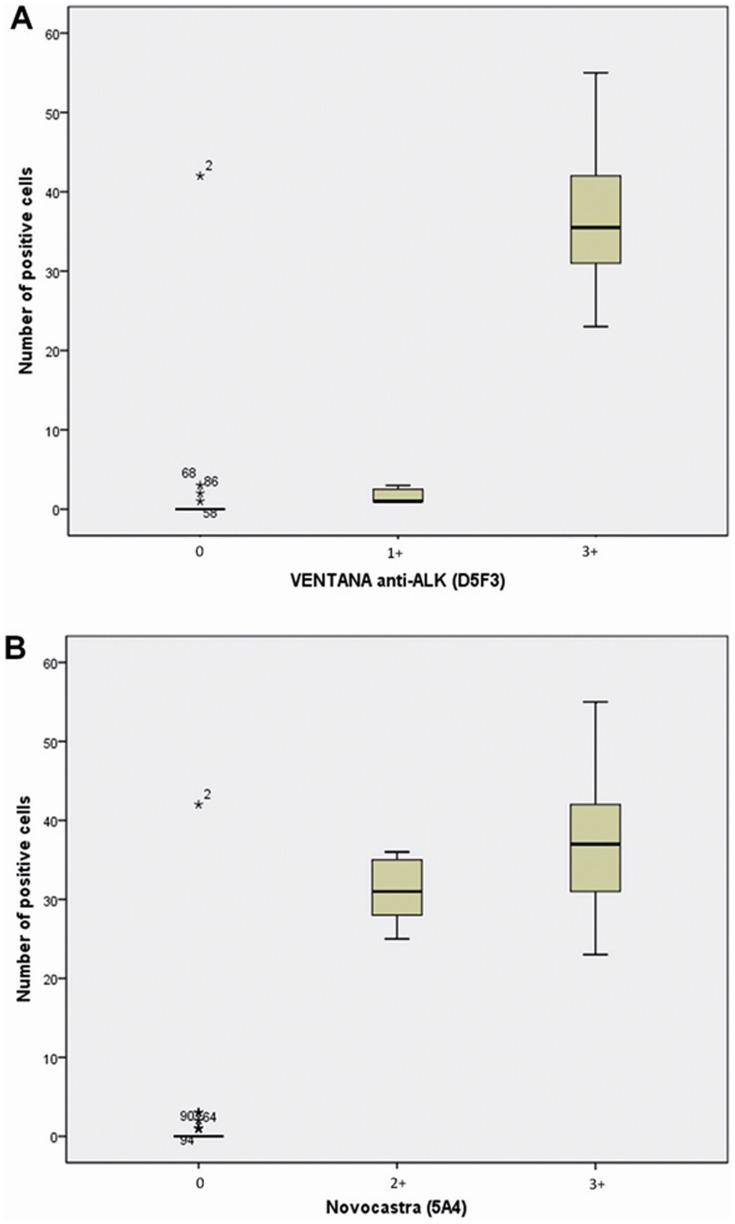
Box plots for number of *ALK* positive cells by FISH automatized technique versus intensity of the ALK IHC staining. With the Ventana anti-ALK antibody (A) and with Novocastra (5A4) antibody (B). Kruskal-Wallis test was performed. The comparisons between the categories in each antibody were statistically significant (p<0,001).

We evaluated the correlation between IHC staining intensity and the number of positive cells by FISH. Increases in the staining intensity by both antibodies were associated with increases in the number of FISH *ALK*-rearranged cells (p<0.001): a staining intensity of 3+ by Ventana IHC resulted in an average of 36.3% FISH *ALK*-positive cells, and a staining intensity of 2+ and 3+ by Novocastra IHC resulted in an average of 31% and 36.9% FISH *ALK*-positive cells, respectively (Figure 3).

### Sensitivity and specificity of ALK IHC and *ALK* FISH

The overall sensitivity and specificity of the IHC in comparison with FISH were 98% and 100%, respectively. The positive and negative predictive value of the IHC was 100% and 98%, respectively.

### Correlation between *ALK* rearrangements and histological data

Among the 47 FISH *ALK*-positive cases, 26 (55.3%) were diagnosed as primary lung origin whereas 21 (44.7%) were metastases from different sites. Of all these samples, nine were bronchoscopic biopsies (19.1%), two core-needle biopsies (4.3%), two cell blocks (4.3%), and 34 surgical resections (72.3%). Interestingly, 50% of the latter were excisions of metastases (n = 17): soft-tissue (n = 10), lymph nodes (n = 6) and ovary (n = 1). Pathological characteristics of the *ALK*-positive tumors were as follows: 43 (91.5%) adenocarcinomas (ACs), one (2.1%) SCC, and three (6.4%) NSCLCs NOS. Among the ACs, a predominant solid and cribiform pattern was observed in 28 out of 43 (65.1%); 11 (25.6%) cases presented acinar architecture; and four (9.3%) a predominant papillary pattern. Signet ring cells were observed in 21 of 43 (48.8%) positive cases, as previously described [26]–[28].

## Discussion

We have studied one of the largest series of *ALK* positive tumors to date. A review of published reports identifies very few larger series of such tumors investigated by more than one methodology, and two of those correspond to surgically treated early stage tumors [11], [24], [27], [29]–[32]. We find that both IHC and FISH are reasonable approaches for primary routine *ALK* testing, provided that samples have at least 50 informative tumor cells. This is the number of tumor cells that are required for the FDA-approved FISH *ALK* assay. Using this selection criterion, all but one of the FISH positive cases were confirmed with both IHC antibodies. Interestingly, this single IHC false negative result occurred in a patient with a *bona fide* SCC (i.e., lobectomy with a p40 positive tumor by IHC) that had a partial response to crizotinib (data not shown). Although the *ALK* translocation may be found in pure squamous carcinoma of the lung (such as the one reported herein), the role of *ALK* inhibitors in this setting is still controversial [33]. Interestingly, in a recently reported crizotinib phase 3 trial, a very small group of non-adenocarcinoma patients had a remarkable progression-free survival [34]. Taking into consideration the difficulties in determining histologic subtype in small NSCLC biopsies, at present it seems unrealistic to have different *ALK* testing algorithms driven by histology [35]. Nevertheless, histology should always be considered since aberrant ALK expression (i.e., rearrangement negative) has been described in neuroendocrine lung carcinomas [17], [36].

Although the true reason for the discrepancy outlined above remains unclear, there are two main possible explanations: (a) biological, *ALK* variant-related [12] or due to heterogeneity of staining, as this situation has been reported specially in SCC and adenosquamous carcinoma [13], [37]; and, (b) methodological, due to suboptimal pre-analytical or analytical phases as less sensitive detection systems may result in heterogeneous staining patterns [18]. In this regard, FISH is less affected by the unavoidable variability of the pre-analytical phase in pathology laboratories worldwide, as long as buffered formalin is used as the fixative. Along these lines, there is always a risk of IHC false negatives due to the lack of an *in situ* performance control, as opposed to FISH. External positive controls should not be used to distinguish a negative result from a false-negative result caused by uncontrolled pre-analytical parameters. An interesting comparison can be made with polymerase chain reaction controls. In this methodology, positive control, negative control, water control (equivalent to the negative control in the Ventana assay) and inhibition control or housekeeping gene control (which is lacking in the ALK IHC assays) should be used. Accordingly, we believe that ideally all IHC negative cases should be confirmed by FISH. One may still argue that a single false negative sample is insufficient for this recommendation. However, a careful review of previous studies suggests that our experience is not unique [9], [13], [17], [29], [32], [38]–[43]. Remarkably, in some of these studies *ALK* testing was part of routine clinical care, as in our series. A very recent two-site comparison shows around 30% of FISH positive-IHC negative cases [29]. If using this ultrasensitive IHC approach as a screening tool, a practical recommendation would be to confirm by FISH at least some of the negative IHC results (for example, samples with uncontrolled pre-analytical parameters or with higher probability of harboring *ALK* translocations).

Conversely, the specificity of these ultrasensitive IHC assays [14] obviates the need for FISH confirmation in positive IHC cases. In fact, there have been reports of dramatic responses to crizotinib in patients with IHC positive and FISH negative tumors [44]. From a practical point of view, it is important to bear in mind that in many regions of the world the use of *ALK* inhibitors may not be linked to a specific methodology [2]. Taking into consideration the use of improved IHC protocols, eventual false-positive IHC results are more likely to be an interpretative error rather than a technical error, as has been the case in breast *HER2* testing [45]. Because dichotomous scoring has been shown to enhance reproducibility, we must insist in defining such criteria for each clone. For 5A4, any immunostaining was scored as positive. For Ventana, only weak cytoplasmic staining was considered negative (Figure 2). However, several issues may preclude the use of IHC as a final predictive test. Firstly, the common perception that IHC should be used as a screening test, followed by confirmation of the positive cases with the gold-standard method. The proposed algorithm for the use of mutation-specific EGFR IHC has been a step forward for this change of paradigm [46]. Secondly, there is a lack of inter-laboratory and inter-observer uniformity in assay performance and assay interpretation. In this regard, the standardization of the Ventana assay, from both the analytical and post-analytical point of view, can help implement this strategy. Our results with the Novocastra antibody and the ultrasensitive IHC protocol are very similar to those of other groups [47].

Finally, it must be emphasized that we (E.C, unpublished data) and others [14], [48], [49] have found positive ALK IHC particularly useful in limited samples or when FISH is not evaluable. However, a broadly held consensus on the number of positive cells required for an IHC positive score has yet to emerge. Indeed, it has been shown that, when less than 50 tumor cells are present, there is a risk for false-negative IHC results [9]. Accordingly, the number of IHC positive cells has been compared with staining intensity, for example, a staining intensity of 2+ required 58.2% of positively stained cells [50]. The significant correlation that we found when we compared the number of FISH positive cells and the IHC intensity further supports the validity of our data.

Due to a series of factors which often coexist, it is difficult to apply the findings of *ALK* testing published in the literature to the clinical reality. Outside of clinical trials or referral testing laboratories [29], [34], [51], most series mainly test surgically resected specimens or tissue microarrays [11]–[13], [16], [27], [50], [52]–[59] rather than small biopsies with intention to treat [9], [14], [30], [31], [38], [60]–[62]. Therefore, one of the strengths of this study is that this large cohort of *ALK* positive samples was initially tested with intention to treat. However, the fact that over 72% of the samples were “large” specimens (50% of them surgically resected metastases) is a minor limitation of our series and may not represent routine clinical practice. Moreover, we had very few cytology samples which are the most common form of diagnostic material in many institutions. Although recently released guidelines [35] recommend the use of cell blocks, excellent results have been reported for both IHC and FISH with stained smears and liquid-based preparations [14], [38], [63]. Another potential caveat of our work is that this is a retrospective series and we cannot comment on the performance of the assays in predicting response to *ALK* inhibition. To partially overcome this shortcoming, we decided to increase the robustness of the gold standard. Reasoning that the *ALK* FISH assay is especially difficult to interpret and prone to both false-negatives and false-positives [9], [14], [32], [38], [49], [59], [64], we used an outstanding automated FISH scanning system that has recently received FDA-clearance. This strategy provided fast automated scanning, which reduced overall scoring and reporting time, provided standardization of the FISH signal interpretation and ensured sensitive counting.

In summary, we find that IHC and FISH techniques are optimal for the detection of *ALK* translocations in NSCLC patients if at least 50 tumor cells are scored and protocols are strictly followed. The interpretative stringency provided by using negative controls and knowledge of interpretation patterns can avoid IHC false positive cases. The real-world likelihood of false negative IHC results, whether biological or methodological, strengthens the case for FISH confirmation, at least in some situations (for example, in samples with uncontrolled pre-analytical parameters or with higher probability of harboring *ALK* translocations). A consideration of the clinical problem of NSCLC highlights the need to be aware of how the methods that we use perform in reality.
